# Controlling the topology of mammalian mitochondrial DNA

**DOI:** 10.1098/rsob.210168

**Published:** 2021-09-22

**Authors:** Katja E. Menger, Alejandro Rodríguez-Luis, James Chapman, Thomas J. Nicholls

**Affiliations:** Wellcome Centre for Mitochondrial Research, Biosciences Institute, Newcastle University, Framlington Place, Newcastle upon Tyne NE2 4HH, UK

**Keywords:** mitochondria, mitochondrial DNA, topoisomerases, mitochondrial disease, DNA topology

## Abstract

The genome of mitochondria, called mtDNA, is a small circular DNA molecule present at thousands of copies per human cell. MtDNA is packaged into nucleoprotein complexes called nucleoids, and the density of mtDNA packaging affects mitochondrial gene expression. Genetic processes such as transcription, DNA replication and DNA packaging alter DNA topology, and these topological problems are solved by a family of enzymes called topoisomerases. Within mitochondria, topoisomerases are involved firstly in the regulation of mtDNA supercoiling and secondly in disentangling interlinked mtDNA molecules following mtDNA replication. The loss of mitochondrial topoisomerase activity leads to defects in mitochondrial function, and variants in the dual-localized type IA topoisomerase TOP3A have also been reported to cause human mitochondrial disease. We review the current knowledge on processes that alter mtDNA topology, how mtDNA topology is modulated by the action of topoisomerases, and the consequences of altered mtDNA topology for mitochondrial function and human health.

## Graphical abstract

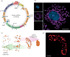

## 1. Introduction

The mitochondria of eukaryotic cells are the product of an ancient endosymbiotic merger between an alpha-proteobacterium and a host cell [1]. While the original bacterial endosymbiont may have been expected to possess several thousand genes [2], a process of gene loss, and the transfer of genes to the nucleus, means that mitochondria are no longer functionally independent. Nevertheless, mitochondria retain a small and highly reduced vestige of the original bacterial genome, called mitochondrial DNA or mtDNA. Mitochondria are the site of a number of essential cellular processes, including oxidative phosphorylation (OXPHOS), fatty acid oxidation, and the biosynthesis of iron–sulfur clusters and haem. The majority of cellular energy, in the form of ATP, is generated via OXPHOS, which is carried out by five large multi-subunit protein complexes at the inner mitochondrial membrane (IMM). All of the protein products of mammalian mtDNA are components of the OXPHOS machinery, with mtDNA-encoded genes contributing 13 of the approximately 90 proteins that constitute the OXPHOS complexes [3]. The human mitochondrial genome is a double-stranded, circular, multicopy DNA molecule. A human cell contains between several hundred and several thousand copies of mtDNA, dispersed within the cellular mitochondrial network [4]. A loss of mtDNA function (resulting from mutations or deletions in mtDNA), or an inability to maintain a sufficient number of copies of mtDNA per cell (termed mtDNA depletion), cause a sub-group of human mitochondrial diseases. The close association between mitochondria and cellular energy production means that these disorders commonly manifest in tissues with high metabolic demand, such as the brain and muscle [5].

The current best estimate for the number of proteins that localize to mitochondria in human cells is approximately 1100 [6,7]. Aside from the 13 mtDNA-encoded genes of the OXPHOS complexes, all mitochondrially localized proteins are therefore encoded in the nucleus and must be targeted to mitochondria and post-translationally imported via a specialized import machinery [8]. The mitochondrial genome is therefore under nuclear genetic control, with an estimated 250–300 nuclear-encoded proteins being required for mtDNA expression [9].

All transactions between proteins and DNA sequences cause alterations to the topology of the DNA. As with any B-form dsDNA molecule, mtDNA adopts a right-handed double-helical structure, with the genetic information being contained at the centre of this helix. In order to replicate, repair or transcribe mtDNA, interacting proteins must manipulate the structure of the DNA molecule in order to gain access to the genetic sequence. Conversely, the packaging of mtDNA by proteins also alters the topology of mtDNA, and consequently also the accessibility of sequence elements required for transcription and replication [10]. The manipulation of DNA topology is generally considered to be essential for genome function, for example for activating transcription [11], but also creates possibilities for genome instability [12]. Mechanisms are therefore required to maintain the topological state of DNA, and a family of enzymes called topoisomerases is involved in maintaining proper DNA topology in all domains of life [13].

In the case of mtDNA, defects in either mtDNA replication or transcription manifest as the impairment of OXPHOS and mitochondrial dysfunction, which may lead to mitochondrial disease. In this review, we explore the factors that impact upon mtDNA topology, the mechanisms that regulate this topology and the consequences when these mechanisms are dysfunctional.

## 2. Structure and function of mitochondrial DNA

**Figure 1.**
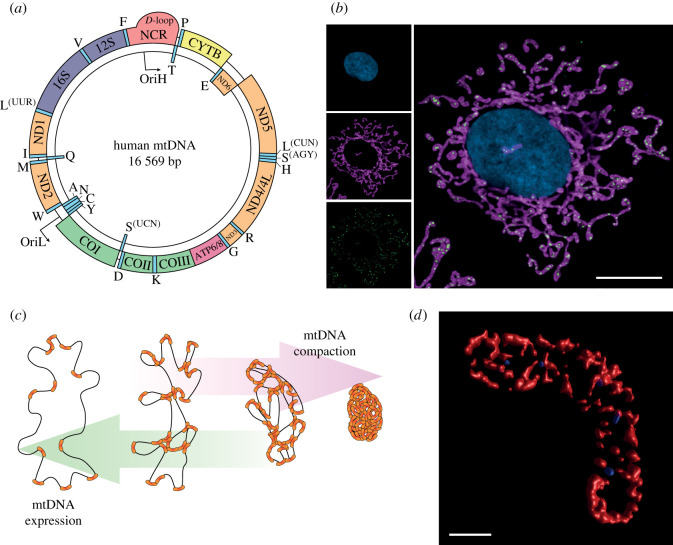
mtDNA structure, distribution and packaging. (*a*) Map of human mitochondrial DNA. The loci of genes encoded on the L-strand (inner circle) and the H-strand (outer circle) are indicated. (*b*) Distribution of mtDNA nucleoids within a human cell. Representative super-resolution Airyscan image of a HeLa cell, with the mitochondrial network labelled using an antibody against the outer membrane protein TOM20 (magenta), mtDNA nucleoids labelled with an anti-DNA antibody (green) and the nucleus stained using DAPI (blue). Merged and single channels are shown. Scale bar represents 10 µm. (*c*) Packaging of mtDNA by TFAM. The binding of TFAM bends and compacts mtDNA to form the nucleoid. Greater concentrations of TFAM create increasingly compacted nucleoids that are unable to undergo transcription and replication. (*d*) Three-dimensional rendered super-resolution microscopy image of packaged mtDNA nucleoids within a mitochondrion. A three-dimensional cross-section of TOM20 (red) and mtDNA nucleoids (blue) was acquired using STED microscopy in a HeLa cell. To visualize the nucleoids as three-dimensional objects, the acquired z-stack was deconvolved and rendered using the surface render function within the Huygens Essential software package. Scale bar represents 0.5 µm.

Human mtDNA is a 16 569 bp circular dsDNA molecule with genes encoded on both strands [14,15]. In addition to encoding protein products of the OXPHOS machinery, mtDNA also encodes all of the RNA molecules required for the expression of these proteins, consisting of a minimal set of 22 tRNAs and two ribosomal RNAs (figure 1*a*). The two strands of mtDNA are designated as the ‘heavy’ and ‘light’ strands, as their differing guanine contents confer different buoyancies during alkaline CsCl_2_ density gradient centrifugation [16].

Similar to its bacterial ancestors, mtDNA is highly compact and does not contain introns. Human mtDNA possesses only one major non-coding region (NCR), which contains a number of sequence elements that are required for the replication and expression of mtDNA. This includes the promoters for mitochondrial DNA transcription, termed the light-strand promoter (LSP) and the heavy-strand promoter (HSP), and the origin of replication for the heavy strand (OriH) [17]. The origin of replication for the light strand, OriL, is found within a small separate NCR within a cluster of tRNAs. The two replication origins, OriH and OriL, are sometimes thought of as dividing the mtDNA into two unequal parts, termed the major arc and the minor arc. In the NCR of a proportion of mtDNA molecules, a 650 nt piece of single-stranded DNA is stably incorporated to form a D-loop structure [18,19]. The proportion of mtDNA molecules containing a D-loop has been found to range from around 10% to around 90%, depending upon the cell or tissue type analysed [18,20–25]. The additional linear strand of the D-loop is called 7S DNA and spans the region between multiple sites close to OriH (at the 5′ end of 7S DNA) and the termination-associated sequence (TAS) close to the gene for mt-tRNA^Pro^ (at the 3′ end of 7S DNA) [26]. The fact that the 5′ ends of 7S DNA are coincident with OriH [27–29] has suggested that 7S DNA may represent either an abortive replication product or a replication primer [30]. However, the D-loop is rapidly turned over, with a half-life of approximately 1 h, and around 95% of replication initiation events result in 7S DNA synthesis rather than full-length mtDNA replication [31,32]. A clear precursor–product relationship between 7S DNA and replication of full-length mtDNA has been elusive, and so the exact reason why the D-loop is maintained and turned over, at significant energetic cost, remains unclear [33].

Unlike nuclear gene expression, protein synthesis in mitochondria is not compartmentalized, with transcription and translation both taking place within the mitochondrial matrix. Mitochondrial transcripts are processed within structures termed RNA granules [34], found adjacent to the mtDNA, and their protein products are embedded directly into the IMM by the mitoribosome during translation [35–37]. The ability of mtDNA to diffuse freely around the mitochondrial network is limited [38]. Mitochondrial dynamics, that is, fission and fusion of the mitochondrial network, is required in order to facilitate the distribution of mtDNA within the cell [39–41]. The mitochondrial function of a cell can therefore be affected either by a depletion in the number of mtDNA molecules, or by a loss of mtDNA stability (in the form of deletions or mutations) in a subset of the mtDNA molecules within a cell [5].

## 3. Topological considerations for mtDNA

Despite being a circular genome of bacterial origin, mtDNA has a number of unusual features that confounds simple comparisons with bacterial mechanisms of DNA topology control. Many of the proteins that replicate and transcribe mtDNA are homologous to bacteriophage proteins, rather than to bacterial proteins. This includes the mitochondrial DNA polymerase catalytic subunit POLGA, the replicative helicase TWINKLE and the RNA polymerase POLRMT, all of which show homology to proteins of the T-odd lineage of bacteriophages [42,43]. The T7 DNA polymerase uses the *E. coli* host thioredoxin as an accessory factor [44], a role that is functionally replaced by the accessory factor POLGB in mitochondria [45,46]. Other essential proteins of the mtDNA replication machinery, such as mitochondrial single-stranded DNA-binding protein (mtSSB) and topoisomerases, are either of bacterial origin or shared with the nucleus [47–49], as may be predicted from the endosymbiotic theory. Mitochondria operate an unusual asynchronous method of DNA replication [50,51] and do not appear to possess a machinery and mechanism for homologous recombination [52], both of which have implications for mtDNA topology control. Topological domains within mtDNA, if present, would presumably be determined by the orientation of transcription units and replication origins, and by potential interactions with the IMM, and will be discussed in the following sections.

### 3.1. mtDNA packaging

Like nuclear and bacterial chromosomes, mitochondrial DNA is packaged into an ordered nucleoprotein complex that, by analogy to bacterial chromosomes, is termed the nucleoid [53]. In microscopy images, nucleoids can be visualized as punctate foci uniformly spread throughout the mitochondrial network (figure 1*b*) [4,54,55]. The more recent application of super-resolution microscopy techniques to mtDNA has determined that the majority of nucleoids contain only a single-mtDNA molecule rather than consisting of mtDNA multimers [56,57], suggesting that mitochondrial genomes act as functionally independent units. Nonetheless multimeric mtDNAs, and more complex junction-containing mtDNA forms, have also been described in cells [58], and particularly in human cardiac tissue, using gel-based methods and electron microscopy [59,60]. The mechanisms and proteins involved in the formation of these multimeric structures remain poorly understood, but may result from particularly high levels of stalled or aberrant mtDNA replication intermediates in the heart.

A large number of proteins that interact with nucleoids have been identified using immunoprecipitation and proximity biotinylation [61–64]. However, the primary nucleoid protein involved in the packaging of mtDNA is the bifunctional mtDNA packaging and transcription factor TFAM (figure 1*c*) [65]. TFAM is abundant enough to entirely coat mtDNA [56,66,67], and ChIP-seq data have indicated that TFAM binds throughout the mitochondrial genome [68]. The binding of TFAM creates sharp U-turns in the DNA both at promoters [69,70] and in a non-sequence specific manner [71,72]. TFAM shows cooperative binding to mtDNA [57,73,74] and cross-strand binding [57] that explains how the mitochondrial genome is compacted from a contour length of around 5 µm, for unbound mtDNA, into a structure with a diameter of 100 nm *in vivo* (figure 1*d*) [56].

It appears likely that the degree of packaging of mtDNA by TFAM acts to regulate transcription and replication activity within mitochondria. The binding of DNA by TFAM in the presence of the mitochondrial type IB topoisomerase TOP1MT induces supercoiling *in vitro* [75], highlighting the topological changes that are created during mtDNA packaging. At physiological TFAM concentrations, nucleoids of different packaging densities can be observed, ranging from small and densely packaged complexes to large and mostly unbound mtDNA [57,76,77]. By reconstituting nucleoid packaging *in vitro* it has been found that a dense packaging of nucleoids inhibits transcription and replication. Although TFAM is required for these processes as an essential transcription factor, high levels of TFAM binding presumably restrict access to the required *cis*-elements. This has led to the suggestion that small changes in the concentration of TFAM could cause large alterations to mtDNA transcription, and thereby act as a method to modulate mitochondrial gene expression and mtDNA replication [76]. Consistent with this idea, nucleoids have been observed using microscopy that are actively undergoing DNA synthesis but show very little TFAM staining [78], suggesting that unpackaged nucleoids could be linked to gene expression and replication while more compact nucleoids are used for mtDNA storage.

### 3.2. Membrane association of mtDNA

The attachment of mtDNA to membrane structures, either at the origins of replication or elsewhere, has consequences for the formation of topological domains, catenated DNA replication products and DNA segregation. Clear associations between mtDNA and the mitochondrial membrane have been described in non-human systems that aid in the segregation of mtDNA following replication. For example, in trypanosomes, the tripartite attachment complex (TAC) spans the double mitochondrial membrane to directly link the kinetoplast DNA (kDNA) to the basal body of the flagellum to drive kDNA segregation [79]. Double membrane-spanning structures have also been described in budding yeast that may link mtDNA replication to segregation of the replicated genome by mitochondrial dynamics [80,81]. Similarly, human mtDNA is also found closely associated with the cristae structure of the IMM [53,82], although the identity of factors responsible, and specific loci for attachment within mtDNA, have been more elusive. An early EM study found a protein complex of membrane derivation to be associated with the OriH region of mtDNA [83], although the proteins involved have not been identified. The localization of the mitochondrial replicative helicase TWINKLE to the IMM suggests that, at least during replication, mtDNA is membrane-bound [84], which would inhibit the formation of interlinks between replicating mtDNA molecules. The structure and lipid composition of the IMM are also important determinants of mtDNA distribution and stability. The study of this relationship is made challenging by the fact that mtDNA-encoded proteins are themselves structural components of the cristae membranes. Nevertheless, mtDNA is found at regions of high cholesterol content [85] and defects in the biogenesis of cholesterol affect the stability of mtDNA [86,87], suggesting a role in mtDNA attachment or maintenance. The link between mtDNA and mitochondrial membrane structure has also been covered in detail elsewhere [88,89].

## 4. Topoisomerases and mitochondria

**Figure 2.**
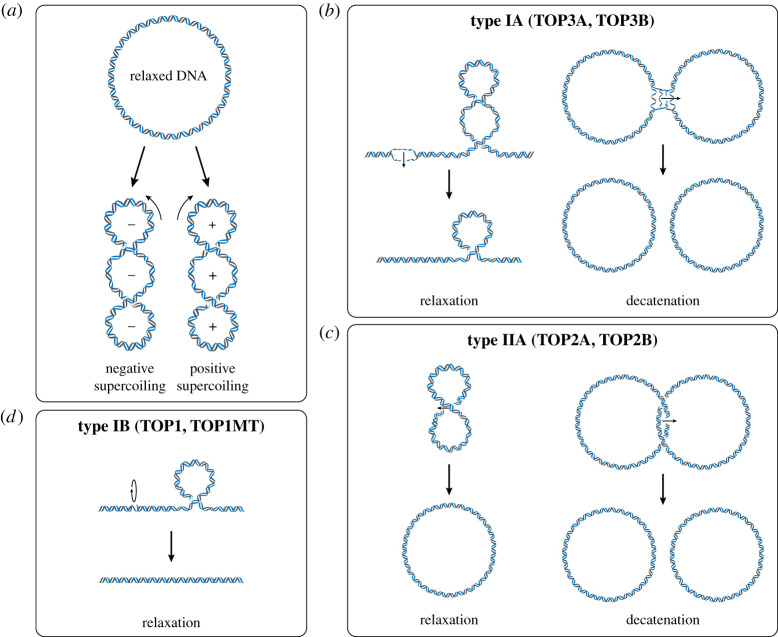
DNA supercoiling and topoisomerase mechanisms. (*a*) DNA supercoiling. A relaxed circular DNA molecule adopts a circular conformation (top). The under-winding of this DNA molecule causes the formation of negative supercoils (lower left), while the over-winding of this DNA causes the formation of positive supercoils (lower right). (*b*) Mechanism of type IA topoisomerases. A transient break is created in ssDNA, an intact strand is passed through the gap and the break is resealed. This strand–passage mechanism permits the removal of negative supercoiling (left) or the decatenation of single-stranded linkages such as hemicatenanes (right) depending upon the structure of the substrate. (*c*) Mechanism of type IIA topoisomerases. A transient break is created in dsDNA, an intact dsDNA strand is passed through the gap, and the break is resealed. This mechanism allows the removal of supercoiling (left) or the decatenation of interlinked dsDNA molecules (right). (*d*) Mechanism of type IB topoisomerases. A nick is introduced into dsDNA, and then the DNA is allowed to undergo a controlled rotation in order to dissipate either positive or negative supercoiling.

In a covalently closed circular DNA molecule such as mtDNA, there are no free DNA ends that can rotate in order to relieve topological strain, and the linking number of the DNA molecule is therefore fixed. In such topologically constrained molecules, transactions between proteins and the DNA duplex create overwound and underwound regions of DNA, called positive and negative supercoils (figure 2*a*). Positive supercoiling represents the tightening of the DNA duplex, and unresolved positive supercoiling will eventually inhibit the progression of DNA and RNA polymerases. Negative supercoiling represents the opening of dsDNA and promotes replication and transcription initiation, as well as the formation of D-loops and other alternative DNA structures [90]. In order to be able to manipulate DNA supercoiling, cells use a family of enzymes called topoisomerases [13]. A topoisomerase creates a transient break in the DNA backbone that can be used to untwist and untangle DNA before the break is re-sealed. Topoisomerases play essential roles in DNA packaging, transcription, DNA replication and recombination [13].

### 4.1. Topoisomerase mechanisms

Topoisomerases are divided into sub-groups depending upon whether they break one strand of DNA (type I) or both strands of dsDNA (type II), with these two types further subdivided according to their reaction mechanism. The human genome encodes six topoisomerases, with two each of type IA (TOP3A and TOP3B), type IB (TOP1 and TOP1MT) and type IIA (TOP2A and TOP2B). Type IA and type IIA enzymes both employ an enzyme-bridged strand-passage mechanism to alter DNA topology. These enzymes create a break in the DNA (in ssDNA for type IA or in dsDNA for type IIA), then an intact DNA strand is passed through the break, and the break is resealed (figure 2*b,c*). If the broken and passaged strands originate from the same DNA molecule then the result is relaxation of the DNA, whereas if the strands are from separate molecules then the result is the linking (catenation) or unlinking (decatenation) of the two molecules [91]. Type IB topoisomerases, on the other hand, act by creating a nick in dsDNA and allowing the nicked strand to undergo a controlled rotation around the intact strand in order to relieve topological tension within the DNA molecule (figure 2*d*) [91]. This mechanism allows type IB topoisomerases to regulate intramolecular supercoiling, while the strand–passage mechanism of type IA and IIA topoisomerases additionally permits a role in the decatenation of interlinked DNA replication intermediates.

### 4.2. The localization and function of human topoisomerases

A prerequisite for a topoisomerase to act upon mtDNA is that it colocalizes with mtDNA in the mitochondrial matrix. The import of proteins into mitochondria is a highly controlled process, as a result of the requirement that the IMM be impermeable to protons in order to carry out OXPHOS. Mitochondrial proteins are directed for import using specialized targeting sequences that can often, but not always, be predicted computationally [8]. Assigning mitochondrial localization to a protein is made challenging by the lack of targeting sequence consensus, and by the fact that targeting sequences are not always located at the N-terminus of the protein. A determination of mitochondrial localization must therefore be made based upon a combination of computational predictions and empirical observation.

The two human type IA topoisomerases, TOP3A and TOP3B, are homologous to the *E. coli* topoisomerase III. TOP3A has a dual localization within mammalian cells, with isoforms targeted to either the nucleus or the mitochondria depending upon the choice of translation start site [40,92,93]. Translation from an upstream start site generates an isoform of TOP3A that bears an N-terminal mitochondrial targeting sequence, whereas translation from a downstream start site generates a shorter isoform of TOP3A that lacks this targeting sequence and localizes to the nucleus [92]. The nuclear form of TOP3A forms a complex together with the OB-fold proteins RMI1 and RMI2, and the RecQ-family helicase BLM, collectively called the BTRR complex [94–96]. This complex is required for the non-crossover resolution (dissolution) of Holliday junctions that arise during DNA recombination [95,97]. Interestingly, nuclear TOP3A has also recently been shown to have the capacity to cooperate with the DNA translocase PICH to introduce positive supercoils [98]. The removal of DNA interlinks by TOP2A is stimulated by positive supercoiling [99], suggesting that the introduction of positive supercoils into interlinked chromosomal DNA by the coordinated action of TOP3A and PICH may act to promote the subsequent removal of these interlinks by TOP2A at the onset of anaphase [98]. However, these binding partners of nuclear TOP3A do not appear to also localize to mitochondria [93], and it is unclear if they are functionally replaced by other factors. The mitochondrial isoform of TOP3A instead appears to be involved in the decatenation of mtDNA molecules during replication, described further in section 6.

TOP3B is unique in being able to process RNA substrates, and localizes to both the nucleus and the cytosol. TOP3B forms a complex with TDRD3 and appears to play a role in the regulation of nuclear transcription, possibly through preventing the formation of R-loops, with the loss of TOP3B resulting in neurological phenotypes [100–103]. Cytosolic TOP3B is found associated with polyribosomes, suggesting that the RNA topoisomerase activity of TOP3B may additionally be required to resolve topological problems with mRNA during translation [104].

The two human type IB topoisomerases, TOP1 and TOP1MT, are expressed from paralogous genes [105]. These two genes have diverged and become specialized for different cellular compartments, with the TOP1 sequence possessing a number of nuclear localization signals that direct it to the nucleus, while TOP1MT encodes an N-terminal mitochondrial targeting sequence that results in its exclusive localization to mitochondria [105,106]. TOP1 has a well-characterized role in regulating supercoiling during nuclear transcription [107,108]. The loss of TOP1MT expression is associated with the dysregulation of mitochondrial transcript levels, suggesting a comparable role for TOP1MT in mitochondria [109,110]. TOP1MT additionally has a proposed role in the regulation of mitochondrial translation [111,112]. TOP1MT is not an essential gene in mice, although knockout animals show alterations to mtDNA supercoiling as well as phenotypes associated with impaired mitochondrial function, also consistent with a role for TOP1MT in mtDNA gene expression [113,114].

The two human type II topoisomerases, TOP2A and TOP2B, play separate roles in nuclear DNA maintenance and expression. TOP2A is only expressed in actively dividing cells [115,116], where it is required for the removal of chromosome interlinks during anaphase [117–119]. TOP2B, alternatively, is constitutively expressed [120] and has a primary function in transcription regulation [115,121]. There is evidence of localization of both TOP2 isoforms to mitochondria in human cells. A number of early studies detected the presence of TOP2 activity in mitochondrial extracts [122–125], while more recent studies have observed mitochondrial localization using cell fractionation, confocal microscopy and mass spectrometry [114,126,127]. Mitochondrially localized TOP2 isoforms have been suggested to be involved in mtDNA replication [48,127] and in the maintenance of the mtDNA D-loop [114]. Our own localization data has not found evidence for either TOP2A or TOP2B in mitochondria [93]. Neither TOP2A nor TOP2B possess a recognizable mitochondrial targeting sequence, and so it remains to be determined if and how these two proteins are targeted for mitochondrial import.

## 5. mtDNA topology during mitochondrial transcription

### 5.1. Transcription units and mechanism

Mitochondrial DNA is transcribed from two promoters, LSP and HSP, which are located close together, and on opposite strands, in the mtDNA NCR [17]. Transcription initiation from LSP also generates a primer for the initiation of mtDNA replication, as discussed in the next section.

Transcription in mitochondria is polycistronic, generating near genome-length transcripts that are cleaved by the endoribonucleases RNaseP [128] and ELAC2 [129,130] to release individual mt-mRNAs, mt-tRNAs and mt-rRNAs [131].

**Figure 3.**
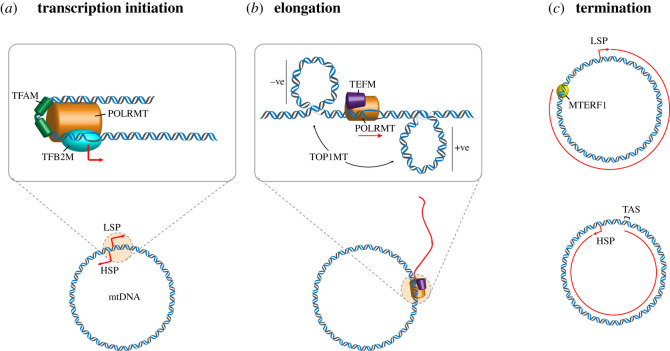
DNA topology during mitochondrial transcription. (*a*) During mitochondrial transcription initiation from LSP and HSP, TFAM binds to the promoter and recruits POLRMT to the TSS. TFB2M is then recruited and facilitates melting of the promoter to permit the initiation of RNA synthesis. (*b*) During transcription elongation, TEFM binds to POLRMT and inhibits the dissociation of POLRMT from the template. TOP1MT regulates the supercoiling state of mtDNA during transcription. (*c*) Transcription initiated at LSP (top) is terminated at the binding site of MTERF1, at the mt-tRNA^Leu(UUR)^ locus. Transcription from HSP (bottom) normally terminates at a conserved sequence element in the TAS region, at the proximal end of the NCR.

A series of elegant biochemical and structural studies have culminated in a model for the initiation of mtDNA transcription from both LSP and HSP. Initiation requires three proteins: the mitochondrial RNA polymerase, POLRMT; and two transcription factors, TFAM and TFB2M [132]. First, TFAM binds upstream of the transcription start site (TSS), inducing a sharp bend in the DNA [69,70], and recruits POLRMT to the TSS [133,134]. TFB2M then binds and aids melting of the promoter DNA [135–137], allowing RNA synthesis to initiate (figure 3*a*). Once the initiation factors have been released, the transcription elongation factor TEFM is recruited to the transcribing polymerase and increases the processivity of POLRMT to allow the synthesis of long polycistronic transcripts (figure 3*b*) [138–141].

Transcription from LSP is believed to be terminated by the protein mTERF1, which binds strongly to the mt-RNA^Leu(UUR)^ locus (figure 3*c*) [142]. mTERF1 exhibits a polarity in its DNA binding, preferentially terminating transcription from the direction of LSP [143–145].

The mechanism of transcription termination for transcripts initiated from HSP remains less well defined, although termination has been observed to occur in the vicinity of TAS (figure 3*c*) [146–148]. The presence of L-strand RNA beyond TAS, spanning the mtDNA control region, has also been described in some circumstances [110,146,148–151].

### 5.2. Control of mtDNA topology during transcription

A moving transcription machinery creates localized regions of positive supercoiling ahead of the RNA polymerase and negative supercoiling behind [152,153]. In a small circular genome such as mtDNA, domains of differential supercoiling can be created either by the anchoring of the DNA to an immobile structure (such as the IMM), or by the presence of transcription units oriented in opposite directions [152]. As discussed in section 3, it remains unclear if and how mtDNA is anchored to the IMM, and so the degree to which mtDNA can freely rotate in order to relieve topological strain is unknown. Because the two mtDNA promoters are located on opposite strands, and oriented in opposite directions, simultaneous transcription from both LSP and HSP would be expected to create domains of positive and negative supercoiling, in front of and behind the two advancing RNA polymerases. However, the close proximity of HSP and LSP initiation complexes [154] suggests the possibility that transcription from the two promoters is coordinated. LSP and HSP also have differing TFAM requirements for activation *in vitro* [132,155]. A clearer picture of the relative regulation of LSP and HSP *in vivo* would enable a better understanding of the topology of mtDNA during transcription.

The mitochondrial type IB topoisomerase, TOP1MT, has been linked to the regulation of mtDNA topology during transcription. TOP1MT physically interacts with POLRMT and has been observed to localize to transcriptionally active nucleoids [109], suggesting that it is involved in removing transcription-associated supercoiling. As a type IB topoisomerase, TOP1MT has the capacity to remove both positive and negative supercoiling and so could act either ahead of, or behind, the mitochondrial transcription machinery. Interestingly, the knockout of TOP1MT is associated with increased negative supercoiling of mtDNA [114], as well as an increased level of mtDNA-encoded transcripts. This suggests that, unless changes to transcript levels are mediated post-transcriptionally, that the action of TOP1MT normally acts to repress transcription [109,110]. TOP1MT knockout mice additionally accumulate non-coding L-strand RNA from the control region, indicative of transcription proceeding past its normal termination site close to TAS [110]. Binding sites for TOP1MT have been mapped to the mtDNA control region, including the promoter region [156,157]. Taken together, these results suggest that the control of mtDNA topology by TOP1MT is normally required to regulate mtDNA transcription initiation and possibly termination.

The potential roles of other mitochondrial topoisomerases in removing transcription-associated supercoiling are yet to be studied, although treatment of cultured cells with compounds known to target TOP2 isoforms has been found to result in a reduction of the steady-state levels of some mitochondrial transcripts [127].

Unresolved supercoiling would eventually be expected to inhibit the progress of the mitochondrial transcription machinery, resulting in premature transcription termination. The tRNA punctuation model dictates that mitochondrial RNAs are synthesized in equimolar ratios as polycistronic transcripts before being processed [131]. The inhibition of transcription progression would therefore lead to a depletion of promoter-distal transcripts; a phenotype that is also seen upon the loss of the transcription elongation factor TEFM [138,141].

## 6. mtDNA topology during DNA replication

### 6.1. Mechanism of mtDNA replication

A small number of core proteins are minimally required to synthesize mtDNA. The mitochondrial DNA polymerase, POL*γ*, is a heterotrimer consisting of one catalytic subunit, POLGA, and two copies of an accessory subunit, POLGB [158]. POL*γ* alone is unable to synthesize DNA using a dsDNA template, and a helicase is required to unwind the dsDNA ahead of the replication fork [159]. The replicative helicase in mitochondria, TWINKLE, forms a hexamer and unwinds dsDNA in the 5′ to 3′ direction [43,160]. Additionally, mtSSB stimulates the processivity of POL*γ* and the helicase activity of TWINKLE [159,161]. A dedicated primase has not been identified for mtDNA replication, and unusually the RNA primers for mtDNA replication are created by the mitochondrial RNA polymerase POLRMT [162,163].

**Figure 4.**
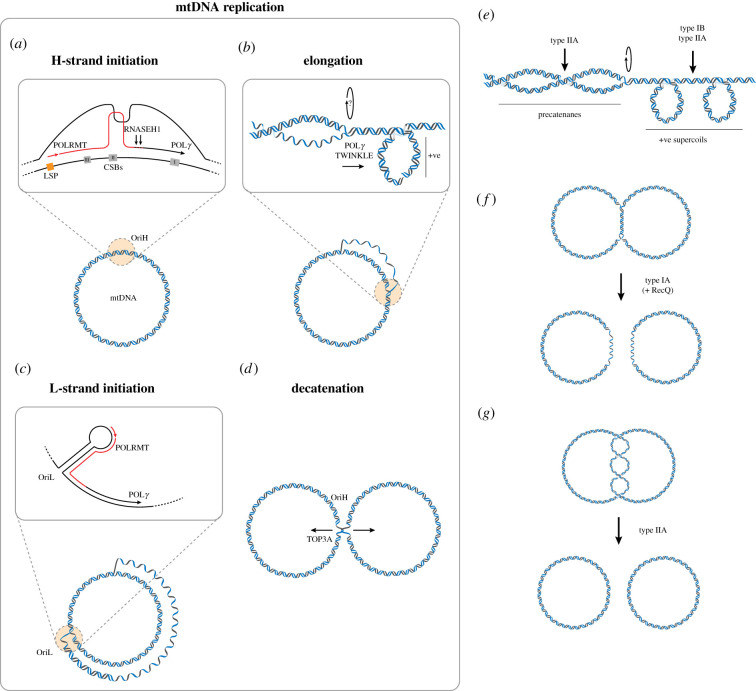
DNA topology during replication. (*a*–*d*) Stages of human mtDNA replication. (*a*) Transcription initiation from LSP creates an R-loop that is stabilized by a hybrid G-quadruplex formed with the non-template H-strand at conserved sequence block II (CSBII). Processing of this R-loop by RNASEH1 creates a primer for the mitochondrial DNA polymerase POL*γ*. (*b*) Mitochondrial DNA synthesis requires POL*γ*, the helicase TWINKLE and mtSSB. The rotation of the replisome to form precatenanes may be inhibited by the binding of TWINKLE to the IMM. (*c*) The primer for L-strand replication initiation at OriL is formed by POLRMT, which synthesizes a short primer from a stem-loop structure in the OriL sequence that can be extended by POL*γ*. (*d*) During mtDNA replication termination, TOP3A is required to separate the replicated mtDNA molecules. In the absence of TOP3A, a hemicatenane forms in the vicinity of OriH, suggesting that this is the primary site of replication termination. (*e*–*g*) DNA topology and decatenation during strand-coupled DNA replication. (*e*) DNA synthesis creates localized regions of positive supercoiling ahead of the replisome, while rotation of the replisome leads to intertwining of the replicated DNA molecules to form precatenanes. (*f*) The convergence of replication forks and the eventual removal of hemicatenanes between replicated DNA molecules can be catalysed by the cooperative action of a type IA topoisomerase (such as TopoIII) and a RecQ-family helicase. (*g*) Precatenanes that remain unresolved following the completion of DNA replication form dsDNA catenanes that require a type II topoisomerase activity for their removal.

Human mtDNA contains two canonical replication origins, oriented in opposite directions, termed OriH and OriL. OriH is located in the NCR, close to LSP, whereas OriL is located in a cluster of tRNAs around 10 kb downstream of OriH. Replication from OriH begins with transcription initiation by POLRMT from LSP, with this transcription terminating in a region of conserved sequence blocks (CSBs) located between LSP and OriH (figure 4*a*). The 3′ end of this primer has been mapped predominantly to CSB II, suggesting that RNA-DNA transitions take place in this region [28,164,165]. CSB II is GC-rich, and the formation of a G-quadruplex structure between the primer RNA and the non-template H-strand produces a stable R-loop [166–168]. The processing of the 3′ end of this R-loop by RNASEH1 generates a 3′ end that can be used by POL*γ* to initiate replication in a reconstituted system [169]. An essential role for mtSSB in directing primer formation during mtDNA replication initiation has also recently been described [170].

Replication initiation from OriL is also primed by POLRMT. In this case, POLRMT recognizes a stem-loop structure that forms in the single-stranded OriL sequence to generate an RNA primer from a poly(T) stretch in the loop of the hairpin [162,163]. Because the formation of this stem-loop requires OriL to be in a single-stranded conformation, leading-strand replication from OriH (figure 4*b*) must reach and displace the OriL sequence before lagging-strand synthesis can be initiated (figure 4*c*) [171,172]. This leads to a substantial delay between replication of the two mtDNA strands.

The exact mechanism of mtDNA replication has been the subject of a substantial amount of debate [51,173,174]. The strand displacement model, formed on the basis of early electron microscopy studies of replicating mouse mtDNA [171], proposes that the displaced parental H-strand is coated with mtSSB during the period between replication initiation at OriH and initiation at OriL. The stem-loop structure at OriL excludes mtSSB from binding at this site, in order to permit primer synthesis by POLRMT [163].

A separate model, known as ‘ribonucleotide incorporation throughout the lagging strand’ (RITOLS), proposes that the displaced H-strand is instead coated by RNA [175–177]. This RNA was later found to be sourced from processed transcripts that are hybridized to the displaced H-strand as the DNA polymerase complex advances, termed the ‘bootlace’ mechanism [178]. A protein machinery for achieving this, as yet, has not been identified.

Fully double-stranded mtDNA replication intermediates, resulting from the simultaneous synthesis of the leading and lagging strands, can also be observed. This strand-coupled mode of mtDNA replication was put forward mostly using evidence from neutral two-dimensional agarose gel electrophoresis (2D-AGE) data, which shows the existence of mtDNA replication intermediates that migrate similarly to dsDNA structures that can be visualized using the same method in yeast and bacteria [179,180].

Due to the asymmetric nature of mtDNA replication, the termination events for mtDNA synthesis take place in different locations for each parental strand. The topoisomerase TOP3A is required for the decatenation of replicated mtDNA molecules (figure 4*d*), as large catenated networks of mtDNA accumulate in the absence of mitochondrial TOP3A activity. Replication termination events have been broadly mapped to the OriH region, suggesting that this is a primary site of mtDNA replication termination [93].

The potential for conflict between replication and transcription also presents a risk for mtDNA topology. Replication initiation from OriH proceeds in the opposite orientation to transcription from HSP, creating the possibility of collisions between the replication and transcription machineries. Whether transcription and replication occur simultaneously within the same mtDNA molecule, beyond the necessity of transcription initiation at LSP to prime replication from OriH remains poorly understood. As noted previously, the proximity of LSP and HSP suggests the possibility that initiation from these two sites is regulated [154]. The mitochondrial transcription termination factor mTERF1, which binds to the mt-tRNA^Leu(UUR)^ locus and preferentially terminates transcription from LSP [143–145] also shows contrahelicase activity that serves to pause mtDNA replication [181,182]. This suggests the possibility that this locus, and the action of mTERF1, could be used to regulate the bypass of replication and transcription complexes.

### 6.2. Control of mtDNA topology during mtDNA replication

#### 6.2.1. Comparison with bacterial and bacteriophage DNA replication

The topological forces acting upon replicating circular genomes, and the action of topoisomerases during replication, is best understood in model bacteria such as *E. coli*. During replication elongation, a moving replisome creates a localized region of positive supercoiling ahead of the replication fork, similar to the situation during transcription. However, compared with transcription, DNA replication bears the added complication of having two nascent daughter DNA molecules behind the replisome, creating the possibility that supercoiling can be dispersed behind the replisome to create intertwines between the two daughter molecules (figure 4*e*) [183]. If left unresolved, these intertwines, termed precatenanes, lead to the formation of catenated daughter genomes during replication termination that necessitates a type II topoisomerase activity for their removal [184–186].

During replication termination, the convergence of two replication forks either generates a hemicatenane that that can be resolved by a type IA topoisomerase such as TopoIII (figure 4*f*), or complete replication of the molecules forms duplex interlinkages that can be removed by a type II topoisomerase (figure 4*g*).

The paradigm of *E. coli* DNA replication indicates that both type I and type II topoisomerases can act to remove chromosome interlinkages during replication elongation and termination. *E. coli* possesses two type II topoisomerases, gyrase and TopoIV, with gyrase acting primarily to regulate supercoiling and TopoIV acting primarily to remove chromosome interlinkages during replication [187,188]. However, TopoIII, a type IA topoisomerase, can also act as a decatenase by acting upon hemicatenanes and single-stranded regions of un-replicated DNA template both *in vitro* and *in vivo* [189–191].

The applicability of this bacterial model to human mitochondrial DNA depends upon both the mechanism of mtDNA replication and the localization of human topoisomerases, in the sense that these factors determine the DNA structures that can be produced and the enzymes present that are capable of processing them.

As previously noted, several proteins of the mtDNA replication machinery are related to bacteriophage proteins of the T-odd lineage [42]. The mitochondrial helicase TWINKLE is related to the T7 gp4 primase-helicase [43], which creates primers for coupled leading- and lagging-strand replication during replication of the phage T7 genome [192]. Replication in T7 results in the formation of linear concatemers that require endonucleolytic processing prior to packaging [193]. By contrast, mitochondrial TWINKLE has lost the primase activity of gp4, with primers instead being synthesized by POLRMT [162,163]. This uncoupling of replication of the two DNA strands may increase the availability of ssDNA regions to act as substrates for type IA topoisomerases.

#### 6.2.2. mtDNA replication initiation and elongation

In strand displacement mtDNA replication, the initiation of mtDNA synthesis from OriH can produce either 7S DNA (to form the D-loop) or initiate full-length mtDNA replication. Pulse labelling studies of replicating mtDNA have suggested that the synthesis of the D-loop removes negative supercoils to produce mtDNA in an open circular form [16,31]. The mitochondrial type IB topoisomerase TOP1MT binds to the mtDNA NCR close to sequence elements that are essential for mtDNA replication, including OriH and TAS at both ends of the D-loop [156,157]. This suggests that TOP1MT could regulate the supercoiling of mtDNA during replication initiation. However, the observation that mtDNA copy number is maintained in the absence of TOP1MT activity [93,114] suggests that this activity would not be essential, or alternatively that it can be performed by another mitochondrial topoisomerase in the absence of TOP1MT. When both TOP1MT and TOP3A are depleted from human cells, severe defects in mtDNA maintenance are observed [93] that may support the idea that TOP3A can compensate for the absence of TOP1MT in the removal of negative supercoiling during mtDNA replication.

During the elongation phase of mtDNA replication, the accumulation of positive supercoiling ahead of the replisome may be expected to promote the rotation of the replisome, resulting in the formation of precatenanes. These precatenanes could be removed by TOP3A, using ssDNA regions in the replicating DNA for strand passage. Precatenanes that are left unresolved following the completion of DNA synthesis would require a type II topoisomerase activity (TOP2A or TOP2B) for their removal. However, an association between the mitochondrial replisome and the mitochondrial inner membrane during mtDNA replication [84] may prevent this rotation of the replisome and therefore inhibit the formation of intertwines between the two daughter mtDNA molecules. In this case, topoisomerase activities would be required to remove localized regions of supercoiling ahead of, and behind, the replisome. Negative supercoiling could be resolved by TOP1MT, TOP3A or a type II topoisomerase, whereas positive supercoiling could only be resolved by TOP1MT or a type II topoisomerase.

#### 6.2.3. mtDNA decatenation

The loss of mitochondrial TOP3A activity is associated with the accumulation of catenated mtDNA replication termination intermediates, centred around the OriH region, indicating a role for TOP3A in mtDNA decatenation [93]. The junctions between these molecules resemble hemicatenanes, consistent with the known catalytic activity of TOP3A [93]. However, an outstanding question is whether this hemicatenated termination structure around OriH represents a physiological intermediate that forms *in vivo*, or whether TOP3A normally acts to unlink mtDNA replication intermediates during replication elongation. As a type IA topoisomerase, TOP3A would require regions of ssDNA in the template DNA in order to remove intertwines between mtDNA replication intermediates. This could be facilitated by long regions of ssDNA present in the lagging strand template, according to the strand displacement model of mtDNA replication, or by short ssDNA regions in strand-coupled replication intermediates. Terminal mtDNA replication intermediates have also been observed around the OriH region using 2D-AGE in wild-type cells containing TOP3A activity [179,194,195], supporting the idea that OriH can act as a replication terminus under normal conditions.

Whether other enzymes also contribute to mtDNA decatenation remains unclear. The nuclear binding partners of TOP3A; the helicase BLM, and the OB-fold proteins RMI1 and RMI2 [94–96,196], have not been observed to localize to mitochondria [93]. The binding of RMI1 to nuclear TOP3A stimulates the decatenation activity of TOP3A both in yeast [197] and in humans [196,198], although it is not absolutely required for this activity. During the binding of RMI1 to TOP3A, a loop from RMI1, termed the decatenation loop, is inserted close to the active site of TOP3A [199]. The decatenation loop is believed to stabilize the gate of TOP3A in a more open conformation and promote its decatenation activity [199,200]. The apparent absence of RMI1 from mitochondria, in which the decatenation activity of TOP3A is essential for mtDNA segregation, therefore seems curious. An interesting observation is that the binding of RMI1 to nuclear TOP3A constrains the size of the gate through which the transferred DNA strand is passed during decatenation [97,199]. It is possible that, in the absence of RMI1, this gate would be large enough to accommodate dsDNA during strand passage, as is the case with *E. coli* TopoIII [201]. If this were the case then mitochondrial TOP3A could act upon a broader range of substrates than currently assumed. For example, TOP3A could potentially decatenate replication intermediates throughout much of the mitochondrial genome during replication elongation, or use the D-loop as a substrate to regulate mtDNA supercoiling.

The cooperation between TOP3 and a RecQ-family helicase (such as BLM) is conserved in both yeast [202] and *E. coli* [203], suggesting a conserved function of the complex. A mitochondrial localization has been reported for the RecQ-family helicase RECQL4 [204–206], and this or another mitochondrial DNA helicase could functionally replace BLM during mtDNA maintenance. Alternatively, it is possible that the lack of homologous recombination within mitochondria to create double Holliday junctions [52], together with an asynchronous mode of DNA replication that avoids the creation of converging dsDNA replication forks, obviates the requirement for a helicase to work together with TOP3A in mitochondria.

A type II topoisomerase could play a role in mtDNA replication either by regulating supercoiling during replication elongation or by decatenating replicated mtDNA, comparable to their roles in the nucleus or in bacteria. Depletion of TOP2B has been found to result in a reduction of mtDNA copy number in one study [127] but not in another [93]. The loss of either TOP2A or TOP2B does not appear to affect the catenation state of mtDNA, arguing against a role of a TOP2 isoform in mtDNA decatenation, but does appear to affect mtDNA supercoiling [93,127]. Drugs that target TOP2 have been observed to affect mtDNA replication rates [127], although it remains to be determined whether this toxicity represents a direct effect within mitochondria mediated via TOP2.

## 7. Topoisomerases in human mitochondrial disease

Missense variants in TOP3A were initially reported in a single individual with an adult-onset mitochondrial disease characterized by progressive external ophthalmoplegia and cerebellar ataxia. On a molecular level, these variants were associated with mtDNA instability (in the form of multiple mtDNA deletions) and the presence of high molecular weight catenated forms of mtDNA [93]. These clinical and molecular features are similar to those associated with some pathological variants in other factors involved in mtDNA replication, such as POL*γ* and TWINKLE [207]. Subsequently, a cohort of ten patients was described with a disorder of growth restriction and microcephaly, associated with truncating variants in TOP3A [208]. This disorder shares a number of features with Bloom syndrome, which is caused by biallelic loss-of-function mutations in BLM, one of the binding partners of TOP3A in the nucleus. However, a number of these patients with truncating variants in TOP3A also exhibited cardiomyopathy, not typically observed in Bloom syndrome, that is likely to be attributable to the loss of activity of the mitochondrial isoform of TOP3A [208]. A further two siblings were also recently reported with a Bloom syndrome-like disorder with cardiomyopathy and mitochondrial dysfunction, resulting from compound heterozygous truncating and missense variants in TOP3A [209]. The relative contributions of the nuclear and mitochondrial isoforms of TOP3A to the clinical features of TOP3A-related disease warrant further investigation.

Variants in TOP1MT are not currently directly implicated in monogenic mitochondrial disease. However, two major single nucleotide variants in TOP1MT have been found to affect the catalytic activity of the protein [210] and could potentially act as modifiers for other variants found in mitochondrial DNA disease.

Compounds that target type II topoisomerases have also been implicated in mitochondrial dysfunction. The chemotherapeutic agent doxorubicin targets both TOP2 isoforms [211], and cardiomyopathy caused by doxorubicin treatment is associated with mitochondrial damage [212]. Other antibiotics such as fluoroquinolones, which are associated with tendonitis in a small number of cases [213], target bacterial type II topoisomerases. Both families of compounds have been suggested to inhibit TOP2 within mitochondria [127], and clarifying the mechanism of action of these drugs and their effects upon mtDNA maintenance is of therapeutic importance.

## 8. Concluding remarks

Maintaining the topological homeostasis of DNA during transcription, replication and packaging is essential for genome stability. In mitochondria, defects in the maintenance of the multicopy mitochondrial genome impact upon the bioenergetic role of mitochondria within the cell and can lead to human mitochondrial disease. Topoisomerases are required both for maintaining mtDNA supercoiling and for mtDNA decatenation and segregation. Molecular roles for two mitochondrial type I topoisomerases, TOP3A and TOP1MT, have been described but further investigation is required to determine the roles of mtDNA topology and packaging in the regulation of mitochondrial gene expression and mtDNA replication. Our future understanding of how mtDNA topology is controlled will be informed by a better understanding of how topological domains in mtDNA are formed during mtDNA expression and replication, and by association with the mitochondrial membrane. In recent years, pathological variants in TOP3A, which has both mitochondrial and nuclear isoforms, have been found to underlie cases of human disease. Understanding the relative contributions of these two isoforms to these disease phenotypes necessitates further study, as does the mechanism of action of drugs that inhibit or poison topoisomerases and result in mitochondrial toxicity.

## Data accessibility

This article does not contain any additional data.
